# Adenosine administration during hybrid atrial fibrillation ablation to test dormant pulmonary vein conduction

**DOI:** 10.1007/s10840-017-0239-5

**Published:** 2017-03-11

**Authors:** Mindy Vroomen, Mark La Meir, Jos G. Maessen, Harry J. Crijns, Laurent Pison

**Affiliations:** 1 Department of Cardiology, Maastricht University Medical Center, PO Box 5800, Maastricht, The Netherlands; 2 Department of Cardiac Surgery and Cardiovascular Research Institute Maastricht, Maastricht University Medical Center, Maastricht, The Netherlands; 3 Department of Cardiac Surgery, University Hospital Brussels, Brussels, Belgium

**Keywords:** Atrial fibrillation, Pulmonary vein isolation, Ablation, Hybrid, Adenosine

## Abstract

**Background:**

Adenosine administration after initial pulmonary vein isolation (PVI) reveals dormant conduction and predicts atrial fibrillation (AF) recurrence. Elimination of dormant conduction when present may increase a long-term success rate of AF ablation procedures. There are no studies till date using adenosine to reveal acute reconduction of pulmonary veins (PVs) after epicardial PVI during a hybrid AF ablation procedure.

**Methods:**

We included 24 patients (21 male, 55 ± 9 years) undergoing hybrid ablation for symptomatic paroxysmal (*n* = 12) and persistent (*n* = 12) AF, using an epicardial bipolar radiofrequency clamp to perform PVI. All antiarrhythmic medications were discontinued 5 days prior to the procedure, except for patients on amiodarone. Thirty minutes after PVI and once sinus rhythm was obtained, a bolus of adenosine (12 to 36 mg) was administered intravenously. The subsequent response was assessed for each PV (*n* = 96) using an *in situ* circular mapping catheter.

**Results:**

Dormant conduction (i.e., the reappearance of PV potentials during at least one beat) was seen in 1 out of 96 PVs (1%). If reconduction was seen, further endocardial ablation using a 3.5-mm irrigated tip catheter was performed until no more reconduction occurred after repeating the adenosine bolus.

**Conclusions:**

Adenosine administration after PVI with the use of an epicardial bipolar radiofrequency clamp in the setting of hybrid AF ablation reveals acute reconduction in 1% of the PVs.

## Introduction

The cornerstone of endocardial catheter ablation procedures for atrial fibrillation (AF) is ablation at the ostium or antrum of the pulmonary veins (PVs), with the endpoint of electrical isolation of these veins from the left atrium (LA). The rationale for this is the seminal observation by Haissaguerre et al. in 1998 that AF was almost always triggered by ectopic beats arising from the muscle sleeves of the PVs [1]. The success rate of PV isolation (PVI) in patients with paroxysmal AF is greater than 80%. One of the most frequent reasons for AF recurrence is PV reconduction [2, 3]. Adenosine may be used following initial PVI to unmask dormant PV conduction [4]. Additional ablation at sites with acute reconnection may improve single-procedure success rates [5, 6].

Although being a common practice during endocardial catheter ablation procedures, there is no data about the safety, feasibility, and efficacy of adenosine administration as a strategy to assess for transient PV reconnection during hybrid thoracoscopic surgical and transvenous catheter ablation procedures for AF.

## Methods

### Patient characteristics

Twenty-four consecutive patients with symptomatic AF underwent hybrid thoracoscopic surgical and transvenous catheter ablation between September 2012 and January 2014. In this period, no stand-alone epicardial ablations, and 258 catheter ablations were performed in the same center. Patients were eligible for a hybrid AF ablation based on one or more of the following criteria: (1) previously failed catheter ablation, (2) failure of at least one antiarrhythmic drug (AAD) class I or III, (3) LA volume ≥29 ml/m^2^, (4) persistent or long-standing persistent AF, or (5) patient preference for a hybrid procedure instead of a percutaneous approach. All patients underwent a transthoracic echocardiography, a cardiac computed tomography, and a pulmonary function test preoperatively. Definitions of paroxysmal, persistent, and long-standing persistent AF; success and failure of ablation; and follow-up monitoring were based on the Heart Rhythm Society, European Heart Rhythm Association, and European Cardiac Arrhythmia Society consensus statement [7].

### Hybrid ablation procedure

The hybrid ablation procedure was undertaken in a manner as described in detail elsewhere [8]. Briefly, one working port and two camera ports were inserted on both sides of the thorax in order to open the pericardium and the transverse and oblique sinuses during selective lung ventilation. Via the femoral venous approach, a His bundle (St. Jude Medical, St. Paul, Minnesota) and a coronary sinus catheter (Medtronic, Minneapolis, Minnesota) were placed under fluoroscopy, and transseptal puncture was performed with a long 8-F sheath (SL0, St. Jude Medical) into the LA, followed by full heparinization. The PVs were mapped and checked with a circular mapping catheter (Lasso, Biosense Webster, Diamond Bar, California). Antral isolation of the right and left PVs as a pair was performed with a bipolar radiofrequency (RF) clamp (AtriCure, West Chester, Ohio). Each application had a duration of about 15 s, with a median output of 10 to 15 W. The endpoint for PV ablation was entrance and exit block, documented by the circular mapping catheter. In case of sinus rhythm after PVI, reinduction of AF was attempted five times by pacing in the coronary sinus for 10 s at the shortest cycle length resulting in 1:1 atrial capture. AF was considered inducible if it lasted more than 1 min. If AF became noninducible, isoproterenol was infused at rates of 10 to 30 μg/min. If AF had not terminated or still was inducible, linear lesions were deployed. A roof line (connecting both superior PVs) and an inferior line (connecting both inferior PVs) were made epicardially using a bipolar RF pen or linear pen device (Isolator Coolrail pen; AtriCure). These two linear lesions, in combination with bilateral antral PVI, result in complete electrical isolation of the posterior LA (box lesion). Any conduction gap in these epicardial linear lesions was ablated endocardially with a 3.5-mm cooled tip RF catheter (Thermo Cool; Biosense Webster). If the patient was known to have typical atrial flutter (AFL) or if this arrhythmia occurred during the procedure, the cavotricuspid isthmus (CTI) was ablated endocardially. If the right atrium was dilated (>64 mL), two additional epicardial ablation lines were placed: one encircling the superior caval vein and the other connecting both caval veins. Patients still in AF after PVI and deployment of linear lesions underwent electrical cardioversion. The left atrial appendage (LAA) was excluded using a clipping device (AtriClip; AtriCure). The indication for LAA exclusion was a CHA_2_DS_2_-VASc score ≥2. The pericardium was approximated with a stitch, and a chest tube was placed in both pleural cavities.

### Adenosine administration

At least 30 min after epicardial PVI and once sinus rhythm was obtained with other ablation lesions and electrical cardioversion if necessary, a Lasso catheter was placed sequentially at the ostium of each PV and in the area of the box. If bidirectional block was still present, a bolus of adenosine (12 mg at least) was given intravenously for each PV. If necessary, this bolus was repeated with a higher dose until at least one blocked P wave occurred or a pause ≥3 s. Dormant conduction was defined as the reappearance of PV potentials in a PV or in the box during at least one beat. The PVs and box were also checked for exit block after adenosine administration. In the event of dormant conduction, selective endocardial ablation at sites of reconduction was performed. After additional endocardial ablation, adenosine administration was repeated.

### Postablation care and follow-up

Low molecular weight heparin was started 6 h after the procedure, and on the second postoperative day, acenocoumarol was reinitiated. Patients restarted as soon as possible their preoperative AAD regimens. Any symptomatic patient not in sinus rhythm was cardioverted before the 3-month follow-up visit. One patient had a pacemaker, which was used for monitoring. The remaining patients underwent 7-day continuous Holter monitoring at 3, 6, 9, and 12 months. If a 7-day Holter monitoring was not available, patients underwent at least 48- or 24-h Holter monitoring. According to the current guidelines [7], success was defined as no episode of AF, AFL, or any atrial tachycardia (AT) lasting more than 30 s off AAD class I or III after the 3-month blanking period. Acenocoumarol and AADs were discontinued if the 6-month monitoring visit confirmed the absence of atrial arrhythmia.

### Statistical analysis

Data were prospectively entered into a database. Statistical analysis was performed using SPSS version 22.0 (SPSS, Inc., Chicago, Illinois). Continuous variables are summarized with means and standard deviations. Any episode of AF, AFL, or AT lasting more than 30 s detected after the 3-month postprocedural period by electrocardiography, pacemaker interrogation, or 7-day, 48-h, or 24-h continuous Holter monitoring performed at 3, 6, 9, and 12 months was considered failure.

## Results

### Perioperative results

Twenty-four patients underwent a hybrid procedure between September 2012 and October 2014. Twelve patients (50%) underwent a prior PVI for AF (at a mean time of 2 years before the hybrid ablation) and four patients (17%) an ablation for AFL. At the beginning of each hybrid procedure, all PVs were tested for exit and entrance block. In case of AF, the PVs were only tested for entrance block. Only one of all 48 PVs was isolated at an antral level in the patients who had a prior PVI for AF. All AADs were stopped 5 days prior to the procedure except for patients on amiodarone. Patients’ baseline characteristics are shown in Table 1. Eleven patients had persistent AF and one long-standing persistent AF. Eleven patients were in AF at the start of the procedure. In all patients, we achieved a bidirectional block of all the PVs with epicardial ablation (at antral level) only. No endocardial touch-up was needed for PVI. For the left-sided PVs, a mean of 7 ± 2 RF applications was performed with a mean duration of 10 ± 4 s. For the right-sided PVs, a mean of 7 ± 1 RF applications was performed with a mean duration of 9 ± 4 s. In one patient, we did not deploy any other lesion because no arrhythmia was inducible. A box lesion was created epicardially in 23 patients. In 14 patients (61%), we were able to demonstrate endocardial entrance and exit block in the box during sinus rhythm. After endocardial touch-up in nine patients (39%), we completed the box lesion. Five patients needed an endocardial touch-up at the roof line and four at both the roof and inferior line. In seven patients, we created a bicaval line epicardially and isolated the superior caval vein. The cavotricuspid isthmus was ablated endocardially in four patients. The LAA was excluded in 12 patients. Four patients (patients #2, #5, #15, and #21) needed electrical cardioversion at the end of the procedure to restore sinus rhythm. All the other patients were in sinus rhythm.

**Table 1:** Patient characteristics

Patient #	Age (years)	Gender	EF (%)	AF type	AF duration (months)	LA volume (ml)/lpsa (mm)	AAD before procedure	BMI (kg/m^2^)	Previous CA	Hypertension	CAD	Lesion set	Follow-up
1	53	M	37	Parox	24	75/38	Amiodarone	32.1	PVI with cryoballoon	No	Yes	PVI, box	1 year: NSR without AAD
2	61	F	52	Pers	240	100/42	Flecainide	39.1	PVI with cryoballoon	Yes	No	PVI, box	1 year: NSR with AAD
3	65	M	58	Pers	132	100/40	None	26	No	No	Yes	PVI, box, bicaval line, SCV, CTI	1 year: NSR without AAD
4	41	M	50	Parox	131	126/48	Flecainide	28.5	PVI with cryoballoon, CTI with cryocatheter	No	No	PVI, box	1 year: AF with AAD
5	54	M	46	Pers	156	99/57	Sotalol	28	CTI with RF	No	Yes	PVI, box, bicaval line, SCV, CTI	1 year: NSR without AAD
6	43	M	55	Parox	24	101/51	None	25.7	No	No	No	PVI, box, bicaval line, SCV	1 year: NSR without AAD
7	49	M	54	Parox	60	83/51	Flecainide, sotalol	24.4	PVI and CTI with RF	No	No	PVI, box, bicaval line, SCV	1 year: NSR without AAD
8	52	M	50	Parox	36	94/52	Flecainide	29	PVI with cryoballoon	No	Yes	PVI, box	1 year: NSR with AAD
9	63	M	68	Parox	84	105/52	Flecainide	24.7	CTI with RF	No	Yes	PVI, box	1 year: NSR without AAD
10	61	M	42	Pers	108	131/51	Amiodarone	26.6	No	No	No	PVI, box, CTI	1 year: NSR without AAD
11	67	M	63	Parox	108	68/45	Amiodarone	30.3	No	Yes	Yes	PVI, box	1 year: NSR without AAD
12	62	M	47	LS Pers	48	75/46	None	28.3	No	Yes	Yes	PVI, box	1 year: NSR without AAD
13	55	M	70	Parox	180	77/38	Sotalol	28.7	PVI with RF	Yes	Yes	PVI, box	1 year: NSR without AAD
14	60	F	50	Pers	60	104/39	Sotalol	26.2	No	Yes	Yes	PVI, box	1 year: NSR without AAD
15	72	F	57	Pers	120	67/41	Flecainide	27	CTI with RF	Yes	No	PVI, box, bicaval line, SCV	1 year: NSR without AAD
16	56	M	60	Parox	24	112/38	None	21.6	PVI with cryoballoon, CTI with RF	No	No	PVI, box, bicaval line, SCV, CTI	1 year: NSR without AAD
17	55	M	46	Pers	24	84/51	None	32.3	PVI with RF	Yes	Yes	PVI, box, bicaval line, SCV	1 year: NSR without AAD
18	49	M	56	Pers	48	53/35	None	31.7	PVI with RF	No	No	PVI, box	1 year: NSR without AAD
19	65	M	65	Parox	72	66/44	Sotalol	25.4	PVI with RF	No	No	PVI	1 year: NSR without AAD
20	38	M	59	Pers	36	78/47	Flecainide	26.3	PVI with cryoballoon	No	Yes	PVI, box	1 year: NSR without AAD
21	60	M	60	Pers	13	84/41	Verapamil	29.3	No	No	No	PVI, box, CFAE	1 year: NSR without AAD
22	46	M	53	Parox	54	100/48	Sotalol	27.7	PVI and CTI with cryoballoon + RF	No	Yes	PVI, box	1 year: NSR without AAD
23	46	M	65	Parox	180	97/51	Verapamil	22.5	CTI with RF	No	Yes	PVI, box	1 year: NSR without AAD
24	40	M	58	Pers	12	97/55	None	30.6	No	No	No	PVI, box, CFAE	1 year: NSR without AAD

*AAD* antiarrhythmic drug, *AF* atrial fibrillation, *BMI* body mass index, *CA* catheter ablation, *CAD* coronary artery disease, *CTI* cavotricuspid isthmus, *EF* ejection fraction, *LA* left atrial, *lpsa* left parasternal axis, *NSR* normal sinus rhythm, *Parox* paroxysmal, *Pers* persistent, *LS Pers* long-standing persistent, *PVI* pulmonary vein isolation, *RF* radiofrequency, *SCV* superior caval vein

### Acute reconnection after adenosine administration

**Fig. 1:**
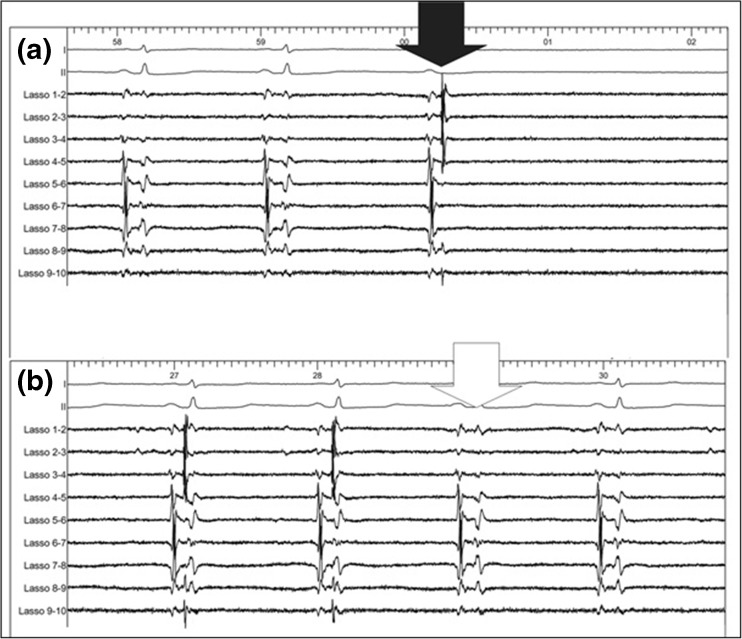
Circular mapping catheter—transient reappearance of PV potentials. **a** The administration of 21 mg of adenosine in patient #14 induced transient AV block and the transient reappearance of PV potentials (*black arrow*) on the circular mapping catheter (Lasso) at the first blocked P wave. **b** Twelve seconds later the PV potentials disappeared (*white arrow*)

**Fig. 2:**
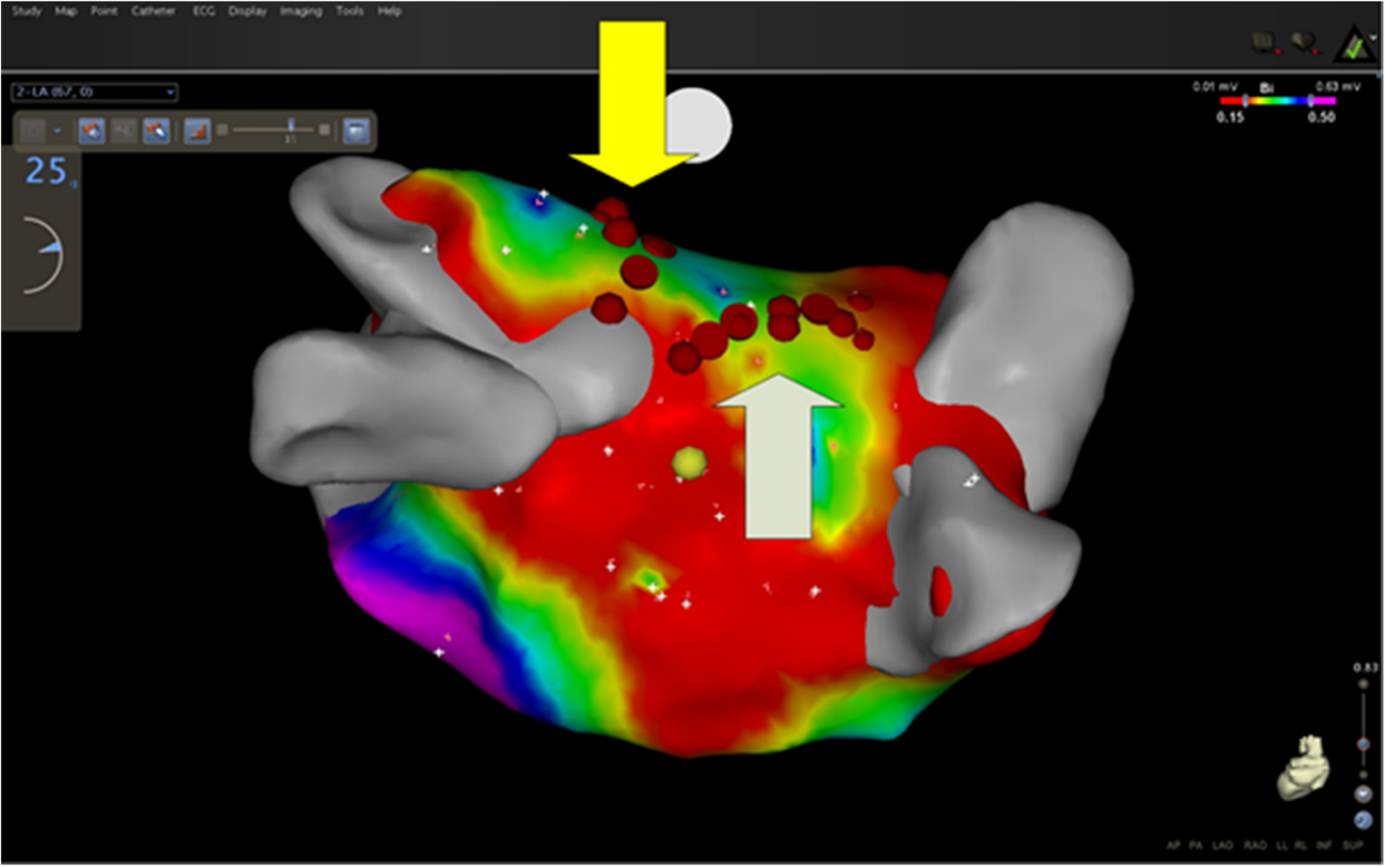
Voltage map—endocardial touch-up. Posterior view of the LA voltage map in patient #14. The RF applications at the junction of the left super superior PV with the roof of the LA are visualized (*yellow arrow*). In this patient, endocardial RF touch-up of the roofline was necessary to complete the box lesion (*white arrow*)

At the time the Lasso catheter was placed at the ostium of the PV, all the PVs (*n* = 96) (24 left superior PVs, 24 left inferior PVs, 24 right superior PVs, and 24 right inferior PVs), and also the area of the box (*n* = 23), still displayed entrance and exit block. In Table 2, the injected doses of adenosine are outlined. Dormant conduction induced by adenosine administration (i.e., the reappearance of PV potentials during at least one beat) was seen in 1 out of 96 PVs (1%) (Fig. 1). It occurred in patient #14 at the junction of the left superior PV ostium with the roof of the LA. Additional endocardial ablations were performed at this site (Fig. 2). Repeating the administration of adenosine did not result anymore in acute reconnection.

**Table 2:** Adenosine dose per pulmonary vein

Left superior PV	17 ± 5 mg (range 12–30)
Left inferior PV	17 ± 6 mg (range 12–36)
Right superior PV	15 ± 3 mg (range 12–21)
Right inferior PV	15 ± 3 mg (range 12–21)

*PV* pulmonary vein

In one case (#13), no entrance block could be verified in the box after the administration of adenosine. The endocardial voltage map showed an incomplete roof and inferior line. After endocardial touch-up, a bidirectional block was achieved.

### Follow-up

All patients (100%) reached 1-year follow-up. At 1 year, 23 out of 24 patients (96%) were in sinus rhythm, with no episodes of AF, AFL, or AT lasting longer than 30 s on office (electrocardiogram) follow-up and Holter monitoring (Table 1). Three patients (12.5%) were still on AADs, of which one had AF, one experienced palpitations which later appeared not to be AF, and one patient suffered AF in the blanking period and wanted to use AAD a few months longer.

### Complications

No deaths or conversion to cardiopulmonary bypass was encountered. No patient demonstrated paralysis of the phrenic nerve. One patient suffered from basilar artery thrombosis after the procedure. The administration of adenosine did not result in any significant hemodynamic instability nor arrhythmias.

## Discussion

This study aimed to test the feasibility and efficacy of administration of adenosine after epicardial ablation in the setting of a hybrid AF ablation. The major findings were as follows:

1. The use of adenosine during hybrid ablation for AF is an effective tool to check for dormant PV conduction at the antral level after epicardial PVI
2. Adenosine reveals dormant conduction at the antral level in only 1% of the PVs after epicardial PVI.

Pulmonary vein reconnection after initial PVI remains the most important reason for AF recurrence in paroxysmal AF [7]. It is present in more than 80% of patients who undergo a repeat procedure [9, 10]. This phenomenon still remains one of the “Achilles heels” of modern invasive electrophysiology. New energy sources and balloon-based devices have been developed to improve long-term results of PVI, but it is too early to draw definitive conclusions [11, 12]. Contact catheter technology is also promising and may provide more long-lasting lesions, but there is no long-term data available yet [13].

Dormant conduction is the phenomenon of transiently restored conduction through a previously isolated PV, induced by an intravenous purinergic agonist such as adenosine. The use of adenosine as a provocative measure to unmask the presence of dormant conduction after PVI was first studied by Arentz et al. in 2004 [5]. They showed that after successful ostial PVI, 25% of the studied PVs regained electrical activity after the administration of adenosine. Since this observation, the use of this drug during PVI procedures became a common clinical practice. Datino et al. described the mechanisms by which adenosine restores dormant PV conduction by recording action potentials from canine LA and PV cells [14]. The restoration of conduction in damaged but viable PVs is based on selective activation of the *I*_KAdo_ inward rectifier current, resulting in hyperpolarization of the resting membrane potential. As PVs with dormant conduction are characterized by less resting membrane depolarization than veins without dormant conduction, the adenosine-induced hyperpolarization will selectively restore excitability in PVs with dormant conduction by removing voltage-dependent *I*_Na_ inactivation.

McLellan et al. published a systematic review on the use of adenosine after PVI [4]. Several interesting observations from this paper are worth highlighting. Data from nonrandomized and retrospective studies showed that patients undergoing adenosine testing and ablation of reconnections had better outcomes than patients in whom adenosine testing was not used. However, in patients with acute reconnection after adenosine and additional ablation at these sites, the occurrence of recurrent AF tended to be higher than in patients without acute reconnection. The reason for this finding remains unclear. A possible explanation could be that acute reconnection is a surrogate marker of the impossibility to create completely transmural circular lesions, e.g., because of anatomic reasons [15]. The ADVICE trial is a prospective and randomized study designed to analyze the effects of additional ablation in PVs, showing acute reconnection after adenosine administration in patients with paroxysmal AF. The recently published results of this trial showed that this strategy unmasked dormant PV conduction in 21% of the tested PVs and that additional adenosine-guided ablation did improve arrhythmia-free survival (69.4 vs. 42.3%) [16]. However, these results could not be confirmed in two also recently published trials in which no significant reduction of recurrence in the adenosine-guided ablation group could be found [17, 18].

Transient reconnection after successful endocardial PVI has been described to occur in up to 35% of the PVs after the use of adenosine [19]. To the best of our knowledge, no study has assessed the effects of adenosine administration after epicardial PVI in a hybrid AF ablation setting, neither in an epicardial setting. In our series of patients, only one single PV (1%) showed dormant conduction. In this particular patient, it was very difficult to guide the bipolar RF clamp completely around the left PVs. The very upper part of the antrum of the left superior PV (junction with the LA roof) never got in between the jaws of the clamp. This might explain the fact why it was not possible to create a completely transmural lesion epicardially at this specific spot and the apparent entrance block of this PV when checking with the endocardial Lasso catheter as a result of incomplete injury. The fact that epicardial PVI seems to result in significantly less dormant conduction might be explained by the greater ability to create completely transmural lesions compared to endocardial energy sources. The epicardial application of bipolar and bilateral RF energy, as used in this series, overcomes the heat sink by clamping the tissue and excluding the effect of the circulating blood on ablation, which seems to result in more persistent lesions [20, 21]. This means that the use of adenosine to reveal dormant conduction might be questioned in epicardial AF ablations.

### Study limitations

The small number of patients in this single-center study prevents definitive conclusions. It remains unclear whether additional endocardial application of RF at sites showing acute reconnection improves long-term results. Prolonging the waiting time after initial epicardial PVI may increase the number of PVs with acute reconnection after adenosine. Although only one of the PVs was isolated in the patients with a previous catheter ablation procedure (tested at the beginning of the hybrid procedure), it cannot be excluded that previous catheter ablation favorably influenced the results. Furthermore, the creation of additional linear lesions (roof and inferior line) connecting with antral PVI may influence the occurrence of acute PV reconnection. The adenosine testing thus must be interpreted in the light of these entire lesion sets, which may have reduced finding dormant conduction. Also, the role of general anesthesia may have played a role in the low prevalence of PV reconnection.

## Conclusion

Adenosine administration after PVI with the use of an epicardial bipolar RF clamp in the setting of hybrid AF ablation unmasks dormant conduction in 1% of the PVs.
